# Supplementary material for: The debranching enzyme Dbr1 regulates lariat turnover and intron splicing
Source: Nat Commun. 2024 May 30;15:4617. doi: 10.1038/s41467-024-48696-1 (PMC11139901; doi:10.1038/s41467-024-48696-1)
Supplement: Supplementary file 1 — Supplementary Information [file 41467_2024_48696_MOESM1_ESM.pdf]

SUPPLEMENTARY INFORMATION

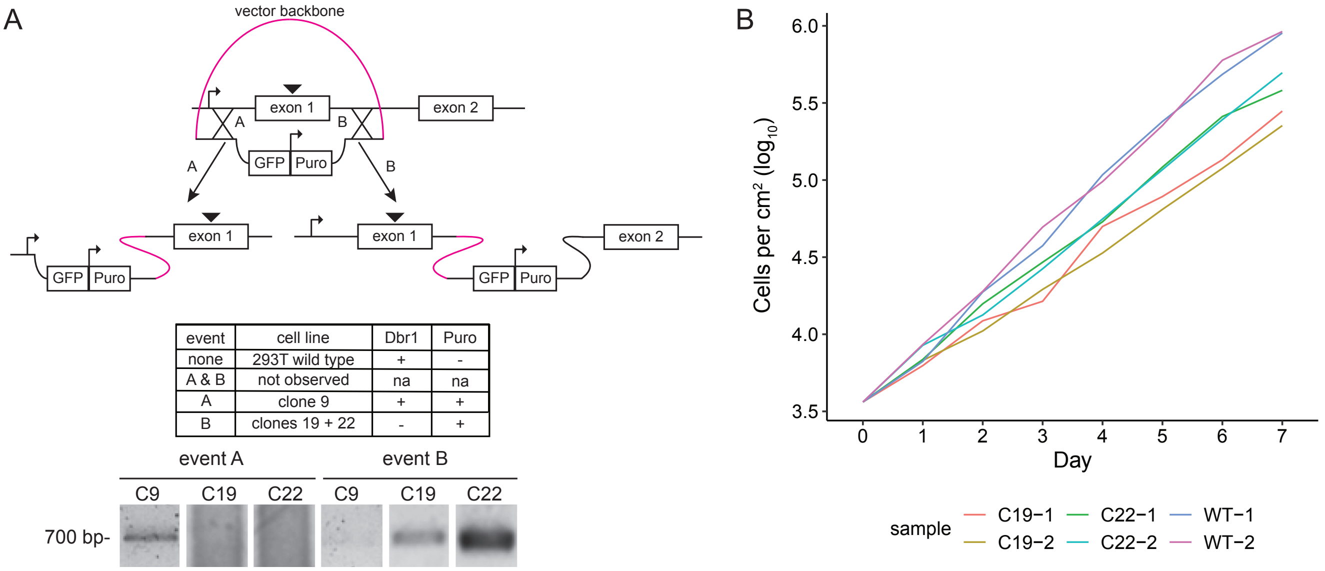

Supplementary Figure 1 – Genomic PCR validation of CRISPR recombination events A) Schematic detailing the potential outcomes of the CRISPR *DBR1* knockout strategy. A diagnostic PCR assay gives a 700 bp band when recombination events ‘a’ and/or ‘b’ occur (bottom). B) Growth curves for wildtype 293T cells and the two *DBR1* knockout cell lines C19 and C22. Source data are provided as a Source Data file.

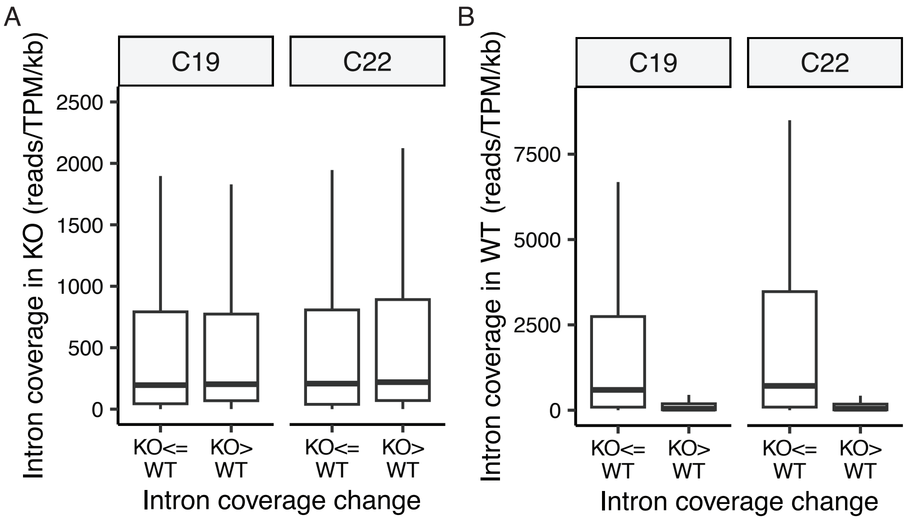

Supplementary Figure 2 – Intron coverage in wildtype and *DBR1* KO A) Normalized coverage in *DBR1* KO samples for introns that decreased (left) or increased (right) in coverage between WT and KO clones C19 and C22. B) A) Normalized coverage in wildtype samples for introns that decreased

(left) or increased (right) in coverage between WT and KO clones C19 and C22. Source data are provided as a Source Data file.

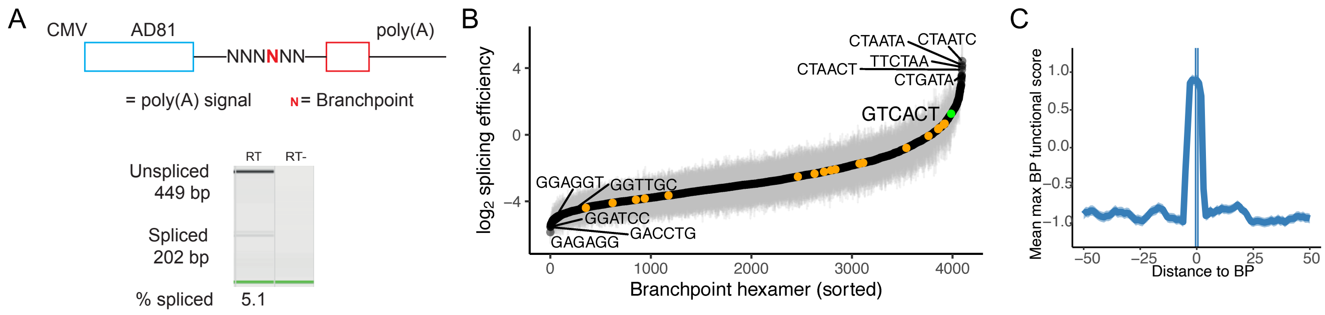

Supplementary Figure 3 – Massively parallel reporter assay measures the ability of any sequence to serve as a branchpoint A) A splicing assay design in which a library of APRT intron 2 sequences with randomized branchpoint hexamers is inserted into a single-intron minigene, and gel of spliced and unspliced products (bottom). B) Splicing efficiencies of the 4096 hexamer branchpoint sequences. Points and grey bars represent mean and ±2 standard deviation of $\log_2$ splicing efficiencies across barcode pairs and replicates. Green point represents wild type branchpoint (GTCACT), and orange points represent branchpoints one mutation away from wild type. Top and bottom 5 branchpoint motifs shown. C) The mean over distance to branchpoint of the max of overlapping hexamer branchpoint functional scores ($\log_2$ splicing efficiency) for each position in a set of introns with only one reported branchpoint. Source data are provided as a Source Data file.

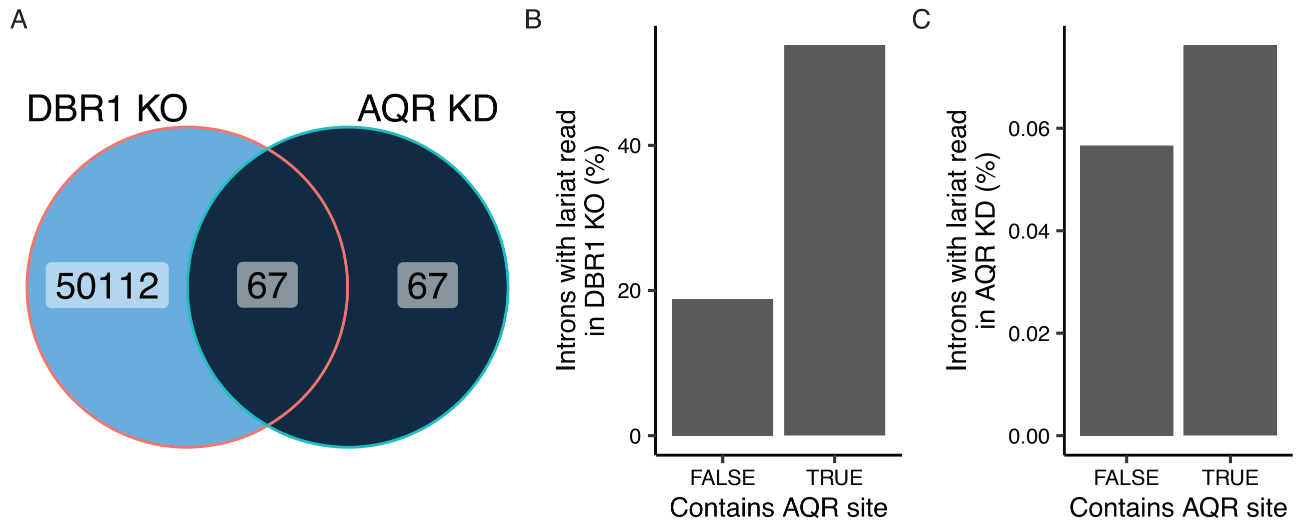

Supplementary Figure 4 – Lariat recovery for introns with and without AQR eCLIP binding sites A) The overlap of introns with lariats recovered in *DBR1* knockout and AQR knockdown samples B) Percentage of introns with a mapped lariat read from *DBR1* knockout samples for the sets of introns with (right) or without (left) a reported AQR binding site. C) Percentage of introns with a mapped lariat read from AQR knockdown samples for the sets of introns with (right) or without (left) a reported AQR binding site. Source data are provided as a Source Data file.

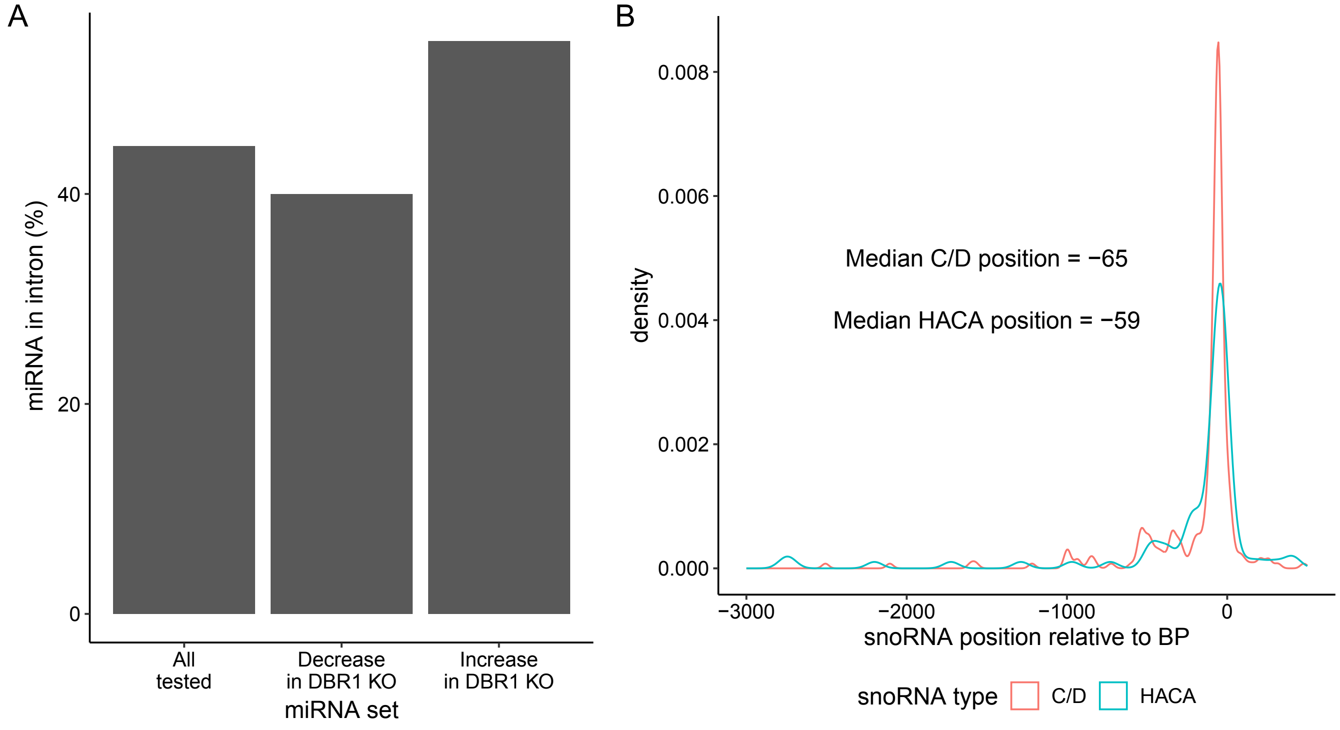

Supplementary Figure 5 – Location of small RNAs expressed in DBR1 KO A) miRNA were tested for differential expression between *DBR1* KO (C19 and C22) and wild type samples. The percent of intronic miRNA was calculated for the sets of miRNA which either decreased or increased in expression relative to wild type. B) The distribution of the positions relative to branchpoints of HACA and C/D snoRNAs expressed in *DBR1* KO samples. Source data are provided as a Source Data file.

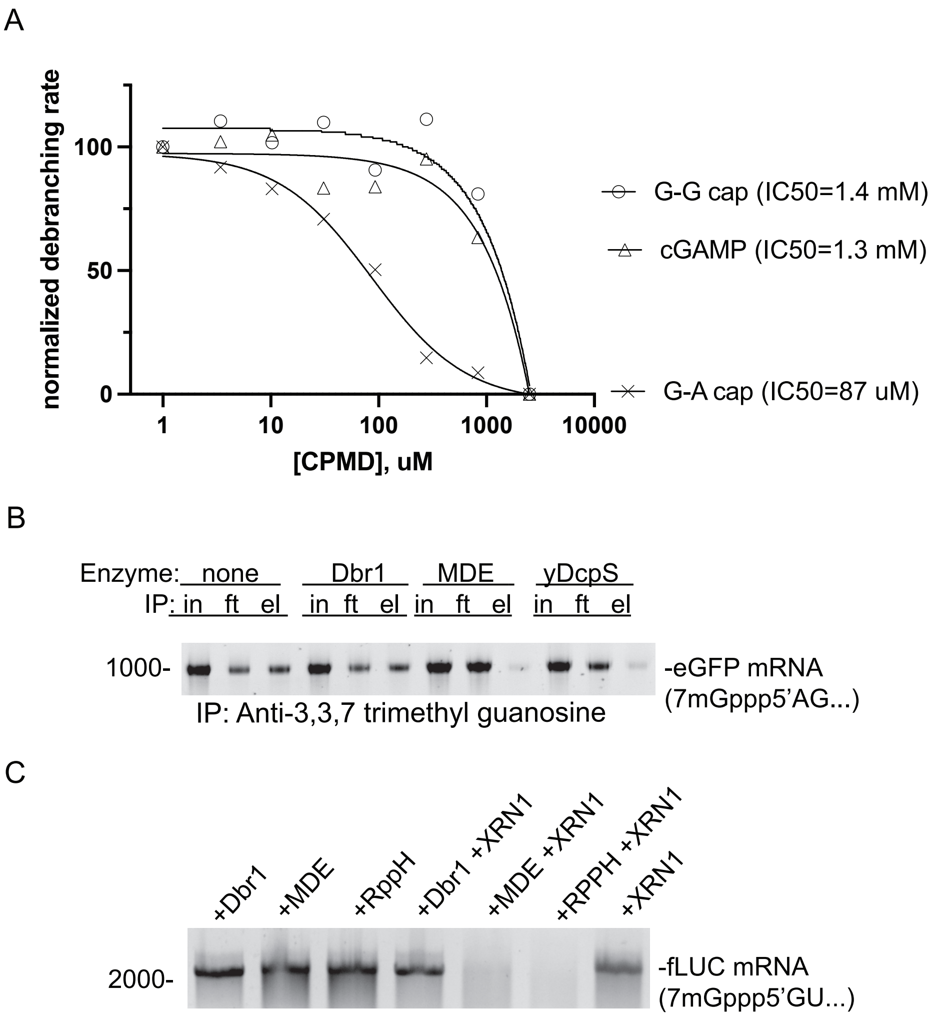

Supplementary Figure 6 – Dbr1 interactions with the 5' mRNA cap A) In vitro inhibition of Dbr1 by G-G and G-A cap analogs or 2'3'-cGAMP. B) Anti-cap immunoprecipitation assay. Capped eGFP mRNA was treated with no enzyme, Dbr1, mRNA decapping enzyme (MDE), or yeast scavenger decapping enzyme (yDcpS), and then incubated with anti-2,2,7 trimethyl guanosine agarose beads, and eluted with excess 7m-G to measure the ability of the enzymes to hydrolyze the cap. C) XRN1 sensitivity assay. Capped firefly luciferase transcript was incubated with Dbr1, MDE, or RPPH, with/without the 5'3' exonuclease XRN1. Cap hydrolysis leads to increased sensitivity to XRN1 degradation. Source data are provided as a Source Data file.

Supplementary Table 1 – Critical Resources Sources and identifiers for the resources used throughout the course of this study.

REAGENT or RESOURCE	SOURCE	IDENTIFIER
Antibodies		
DBR1 (rabbit)	ProteinTech	Cat#16019-1-AP
GAPDH (mouse)	Santa Cruz Biotechnology	Cat#SC-47724
AQR (rabbit)	Bethyl Labs	Cat#A302-546A
HA (mouse)	BioLegend	Cat#901501
Secondary goat anti-mouse Alexa Fluor 647	Thermo Fisher Scientific	Cat#A-21246
Chemicals, peptides, and recombinant proteins		
GeneArt Platinum Cas9 nuclease	Thermo Fisher Scientific	Cat#B25640
Anti-FLAG M2 magnetic beads	Sigma	Cat#M8823
mRNA decapping enzyme	New England BioLabs	Cat#M0608
Yeast scavenger decapping enzyme	New England BioLabs	Cat#M0463
XRN1	New England BioLabs	Cat#M0338
Critical commercial assays		
Minute total protein extraction kit	Invent Biotechnologies	Cat#SD-001
Invitrogen PureLink Genomic miniprep kit	Thermo Fisher Scientific	Cat#K182001
Complete M lysis kit	Roche	Cat#04719964001
Vacinnia capping kit	New England BioLabs	Cat#M2080S
Experimental models: Cell lines		
HEK293T	ATCC	Cat#CRL-3216
HEK293T DBR1 KO	This study	NA
U2OS	ATCC	Cat#HTB-96
Oligonucleotides		
DBR1 sgRNA	5'- AGACGCTGGCGCTGGCAGAG-3'	
Scramble control sgRNA	Origene	
GFP-puro donor DNA	Origene	
Puro (forward)	5'- CCTATGACCGAGTACAAGCCC-3'	
Right homology arm (reverse)	5'-GCGTACTATGGTTGCTTTGACGTATG-3'	
Left homology arm (forward)	5'- CGTAATCATGGTCATAGCTGTTTCCTG-3'	
GFP (reverse)	5'- TAGGTGCCGAAGTGGTAGAAGC-3'	
AK88 bRNA fluorophore	Katolik et al., 2017	
TAOK2 intron 13 Quasar570-labeled smRNA-FISH probes	LGC Biosearch Technologies	Cat#SMF-2006-1
AQR-targeting siRNA s18725	Thermo Fisher Scientific	Cat#4392420
AQR-targeting siRNA s18726	Thermo Fisher Scientific	Cat#4392420
Non-targeting siRNA	Thermo Fisher Scientific	Cat#4390843
7-methylguanosine cap analog	Thermo Fisher Scientific	Cat#AM8048
G-G cap analog	New England BioLabs	Cat#S1407
G-A cap analog	New England BioLabs	Cat#S1406
cGAMP	Invivogen	Cat#nacga23
Recombinant DNA		

Dbr1-FLAG	Zhang et al., 2018	
Software and algorithms		
Split read lariat mapping	https://github.com/jlbuerer/LaMIRA	
Splice site lariat mapping	https://github.com/jlbuerer/lariat_mapping	
Other		
CleanCap EGFP mRNA	TriLink BioTechnologies	Cat#L-7601
