# Supplementary material for: The debranching enzyme Dbr1 regulates lariat turnover and intron splicing
Source: Nat Commun. 2024 May 30;15:4617. doi: 10.1038/s41467-024-48696-1 (PMC11139901; doi:10.1038/s41467-024-48696-1)
Supplement: Supplementary file 3 — Description of Additional Supplementary Information [file 41467_2024_48696_MOESM3_ESM.pdf]

Description of Additional Supplementary Information

Supplementary Data 1 – Dbr1 binding partners. A table of data for each of the 120 enriched interactors in the DBR1 co-IP experiment including information from the Gene-Ontology database (cellular component, biological process and molecular function) as well as spectral counts for the 3 control and 3 co-IP samples.

Supplementary Data 2 – Selected pathways from Gene Ontology and Reactome databases. The list of 120 co-IP MS hits were analyzed with gene set enrichment analysis to find the most significant pathways in Gene Ontology and Reactome databases. Pathways with the lowest FDR values are aggregated in this table. (k) is the number of co-IP hits, and (K) is the total number of entities in the pathway.

Supplementary Data 3 – eCLIP accession Codes. A table providing the accession codes for eCLIP binding peak files from ENCODE. These files can be obtained from the ENCODE website (https://www.encodeproject.org/).

Supplementary Data 4 – Uncropped Gels for Supplementary Figure 1A.

Supplementary Data 5 – Source Data for Supplementary Figure 1B.

Supplementary Data 6 – Source Data for Supplementary Figure 2.

Supplementary Data 7 – Source Data for Supplementary Figure 3B.

Supplementary Data 8 – Source Data for Supplementary Figure 3C.

Supplementary Data 9 – Source Data for Supplementary Figure 4.

Supplementary Data 10 – Source Data for Supplementary Figure 5A.

Supplementary Data 11 – Source Data for Supplementary Figure 5B.

Supplementary Data 12 – Source Data for Supplementary Figure 6A.

Supplementary Data 13 – Uncropped blots for Supplementary Figure 6B/C.
