# Supplementary material for: The debranching enzyme Dbr1 regulates lariat turnover and intron splicing
Source: Nat Commun. 2024 May 30;15:4617. doi: 10.1038/s41467-024-48696-1 (PMC11139901; doi:10.1038/s41467-024-48696-1)
Supplement: Supplementary file 5 — Reporting Summary [file 41467_2024_48696_MOESM5_ESM.pdf]

nature portfolio

Corresponding author(s): William Fairbrother

Last updated by author(s): Apr 17, 2024

Reporting Summary

Nature Portfolio wishes to improve the reproducibility of the work that we publish. This form provides structure for consistency and transparency in reporting. For further information on Nature Portfolio policies, see our Editorial Policies and the Editorial Policy Checklist.

Statistics

For all statistical analyses, confirm that the following items are present in the figure legend, table legend, main text, or Methods section.

n/a	Confirmed	
☐	☒	The exact sample size (*n*) for each experimental group/condition, given as a discrete number and unit of measurement
☒	☐	A statement on whether measurements were taken from distinct samples or whether the same sample was measured repeatedly
☐	☒	The statistical test(s) used AND whether they are one- or two-sided<br>*Only common tests should be described solely by name; describe more complex techniques in the Methods section.*
☒	☐	A description of all covariates tested
☒	☐	A description of any assumptions or corrections, such as tests of normality and adjustment for multiple comparisons
☐	☒	A full description of the statistical parameters including central tendency (e.g. means) or other basic estimates (e.g. regression coefficient) AND variation (e.g. standard deviation) or associated estimates of uncertainty (e.g. confidence intervals)
☐	☒	For null hypothesis testing, the test statistic (e.g. *F*, *t*, *r*) with confidence intervals, effect sizes, degrees of freedom and *P* value noted<br>*Give P values as exact values whenever suitable.*
☒	☐	For Bayesian analysis, information on the choice of priors and Markov chain Monte Carlo settings
☒	☐	For hierarchical and complex designs, identification of the appropriate level for tests and full reporting of outcomes
☒	☐	Estimates of effect sizes (e.g. Cohen's *d*, Pearson's *r*), indicating how they were calculated

Our web collection on statistics for biologists contains articles on many of the points above.

Software and code

Policy information about availability of computer code

Data collection	No custom software was used for data collection.
Data analysis	Lariat mapping was performed with custom code that has been included with the manuscript in a zip file and deposited to Github (accessible at https://github.com/jlbuerer/lariat_mapping and https://github.com/jlbuerer/LaMIRA). Splicing assay results were mapped with STAR (v2.7.10). Alternative splicing analysis was performed by mapping data with STAR (v2.7.10) followed by differential splicing analysis using rMATS (v.4.1.2). Differential gene expression analysis was performed with DESeq2 (v1.24.0). Scaffold v5 was used to process the mass spectrometry data.

For manuscripts utilizing custom algorithms or software that are central to the research but not yet described in published literature, software must be made available to editors and reviewers. We strongly encourage code deposition in a community repository (e.g. GitHub). See the Nature Portfolio guidelines for submitting code & software for further information.

Data

Policy information about availability of data

All manuscripts must include a data availability statement. This statement should provide the following information, where applicable:

- Accession codes, unique identifiers, or web links for publicly available datasets
- A description of any restrictions on data availability
- For clinical datasets or third party data, please ensure that the statement adheres to our policy

Source data are provided with this paper. The RNA-seq data generated in this study have been deposited in the GEO database. DBR1 knockout RNA-seq data is available under accession codes GSE195586 [https://www.ncbi.nlm.nih.gov/geo/query/acc.cgi?acc=GSE195586] and GSE195469 [https://www.ncbi.nlm.nih.gov/geo/query/acc.cgi?acc=GSE195469]. AQR knockdown RNA-seq data is available under accession code GSE195668 [https://www.ncbi.nlm.nih.gov/geo/query/acc.cgi?acc=GSE195668]. The mass spectrometry data generated in this study have been deposited in the MassIVE database under accession code MSV000092263 [https://massive.ucsd.edu/ProteoSAFe/dataset.jsp?accession=MSV000092263]. These data are freely accessible to the public with no conditions. The hg19 genome was used for mapping. eCLIP binding data was obtained from ENCODE. Binding peak files can be accessed through the ENCODE Project website [https://www.encodeproject.org/] using the accession codes provided in Supplementary Data 3.

Research involving human participants, their data, or biological material

Policy information about studies with human participants or human data. See also policy information about sex, gender (identity/presentation), and sexual orientation and race, ethnicity and racism.

Reporting on sex and gender	This study did not involve human participants.
Reporting on race, ethnicity, or other socially relevant groupings	This study did not involve human participants.
Population characteristics	This study did not involve human participants.
Recruitment	This study did not involve human participants.
Ethics oversight	This study did not involve human participants.

Note that full information on the approval of the study protocol must also be provided in the manuscript.

Field-specific reporting

Please select the one below that is the best fit for your research. If you are not sure, read the appropriate sections before making your selection.

☒ Life sciences ☐ Behavioural & social sciences ☐ Ecological, evolutionary & environmental sciences

For a reference copy of the document with all sections, see nature.com/documents/nr-reporting-summary-flat.pdf

Life sciences study design

All studies must disclose on these points even when the disclosure is negative.

Sample size	Statistical methods were not utilized to determine sample size. Sample sizes in this study were selected based on standard practice in molecular biology and are sufficient to assess biological and technical variation.
Data exclusions	No data were excluded from our analyses in this study.
Replication	All important experiments in this study were performed in triplicate, and all attempts at replication were successful.
Randomization	Samples were allocated into experimental groups based on characteristics of the cell line (eg. DBR1 knockout status) or experimental treatment (eg. AQR siRNA treatment). Thus, randomization was not required for the experimental design.
Blinding	This study only involves molecular biology experiments for which there is no group allocation that would make blinding relevant.

Reporting for specific materials, systems and methods

We require information from authors about some types of materials, experimental systems and methods used in many studies. Here, indicate whether each material, system or method listed is relevant to your study. If you are not sure if a list item applies to your research, read the appropriate section before selecting a response.

Materials & experimental systems

n/a	Involved in the study	
☐	☒	Antibodies
☐	☒	Eukaryotic cell lines
☒	☐	Palaeontology and archaeology
☒	☐	Animals and other organisms
☒	☐	Clinical data
☒	☐	Dual use research of concern
☒	☐	Plants

Methods

n/a	Involved in the study	
☒	☐	ChIP-seq
☒	☐	Flow cytometry
☒	☐	MRI-based neuroimaging

Antibodies

Antibodies used

Primary antibodies:
Rabbit Dbr1 (ProteinTech Cat#16019-1-AP)
Mouse GAPDH (Santa Cruz Biotechnology Cat#SC-47724)
Rabbit AQR (Bethyl Labs Cat#A302-546A)
Mouse HA (BioLegend Cat#901501)

Secondary anitbodies:
Goat anti-mouse Alexa Fluor 647 (Thermo Fisher Scientific Cat#A-21246)

Validation

Each antibody was validated by the respective manufacturer for its application, and the validation results are available through the manufacturers' websites.

Eukaryotic cell lines

Policy information about cell lines and Sex and Gender in Research

Cell line source(s)

HEK293T cells (Cat#CRL-3216) and U2OS cells (Cat#HTB-96) were acquired from ATCC. The C22 cell line (DBR1 knockout) was derived from HEK293T cells.

Authentication

The cell lines were not authenticated.

Mycoplasma contamination

The cell lines were not tested for mycoplasma contamination.

Commonly misidentified lines
(See ICLAC register)

No commonly misidentified cell lines were used.

Plants

Seed stocks

Report on the source of all seed stocks or other plant material used. If applicable, state the seed stock centre and catalogue number. If plant specimens were collected from the field, describe the collection location, date and sampling procedures.

Novel plant genotypes

Describe the methods by which all novel plant genotypes were produced. This includes those generated by transgenic approaches, gene editing, chemical/radiation-based mutagenesis and hybridization. For transgenic lines, describe the transformation method, the number of independent lines analyzed and the generation upon which experiments were performed. For gene-edited lines, describe the editor used, the endogenous sequence targeted for editing, the targeting guide RNA sequence (if applicable) and how the editor was applied.

Authentication

Describe any authentication procedures for each seed stock used or novel genotype generated. Describe any experiments used to assess the effect of a mutation and, where applicable, how potential secondary effects (e.g. second site T-DNA insertions, mosiacism, off-target gene editing) were examined.
